# Nontraumatic Parapharyngeal Haematoma: A Rare Lesion

**DOI:** 10.1155/2018/7340937

**Published:** 2018-10-24

**Authors:** Pedro Carneiro de Sousa, Inês Gambôa, Delfim Duarte, Nuno Trigueiros-Cunha

**Affiliations:** ^1^Otolaryngology Medical Resident, Department of Otolaryngology Hospital Pedro Hispano, Unidade Local de Saúde de Matosinhos, EPE, Matosinhos, Portugal; ^2^Otolaryngology Medical Specialist, Department of Otolaryngology Hospital Pedro Hispano, Unidade Local de Saúde de Matosinhos, EPE, Matosinhos, Portugal

## Abstract

Nontraumatic haematoma of parapharyngeal space is very rare and may cause dysphagia and dyspnea. The authors present a case report of a 74-year-old woman with sudden nontraumatic neck swelling without dyspnea and with left pharyngeal bulging and endolaryngeal displacement. Parathyroid hormone elevation and imaging exams confirmed bleeding from a parathyroid adenoma. Symptoms and signs resolved after one week of conservative treatment. There are few cases of parapharyngeal haematomas caused by parathyroid adenomas. Most patients can be managed without emergent surgery, but close airway monitoring is fundamental.

## 1. Introduction

Lesions of parapharyngeal space are rare, comprising only 0.5% of head and neck neoplasms [1]. They may cause dysphagia and dyspnea by compression of upper aerodigestive tract. Nontraumatic rupture of the thyroid arteries is extremely uncommon, with only 40 cases reported until 2002 [2, 3].

Most haematomas resolve spontaneously, so conservative management is often appropriate except in the presence of airway compromise or compression symptoms, when surgical treatment should be considered [1, 3].

## 2. Case Report

**Figure 1:**
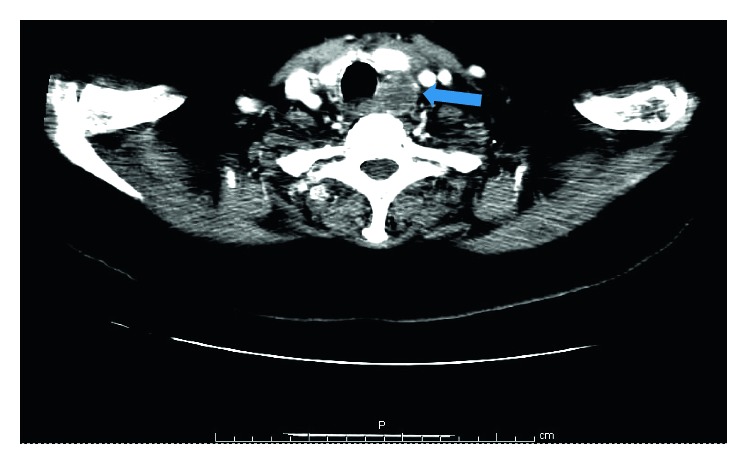
Neck CT showing a left parapharygeal/paralaryngeal nonenhancing mass (blue arrow) with 2.3 cm of maximum diameter.

**Figure 2:**
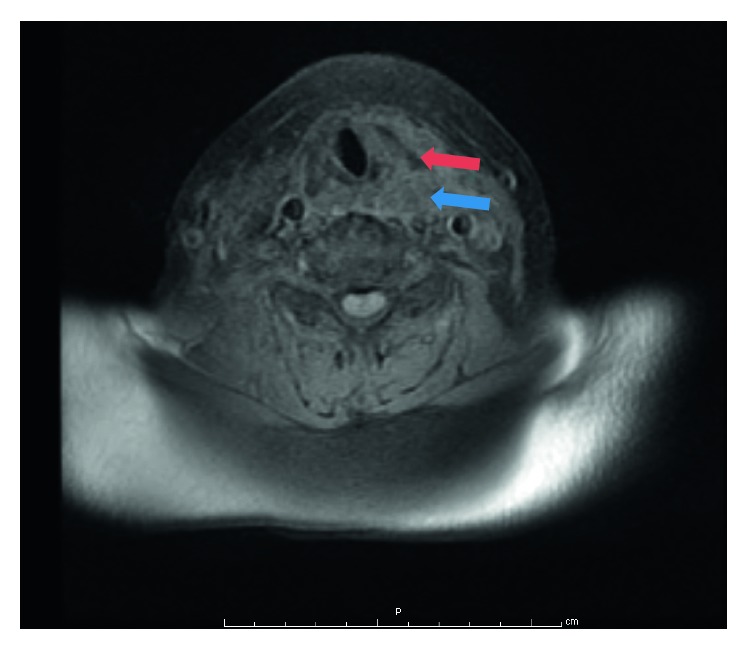
Neck RMI (T1) showing a left capsular lesion posterior to the thyroid gland (blue arrow), with hemorragic laryngeal infiltration (red arrow).

**Figure 3:**
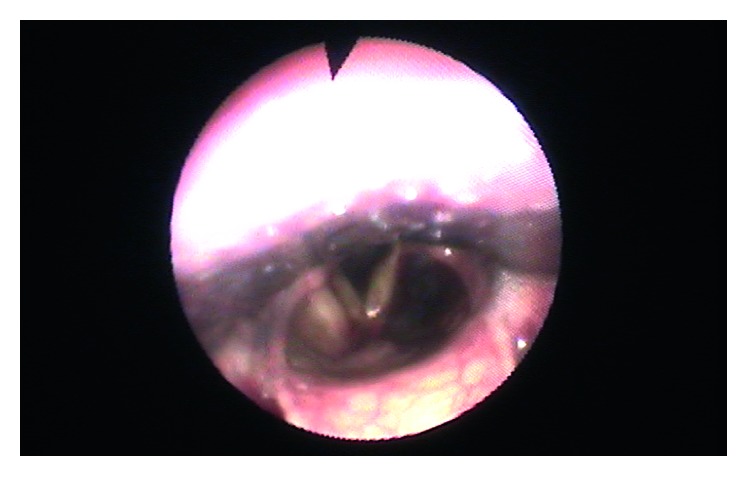
1-week laryngeal fiberoscopy showing normal laryngeal placement and only a slight rosaceous coloration of the left ventricular fold.

A 74-year-old woman presented to the emergency department with sudden onset (24 hours) of painful neck swelling and concurrent dysphonia and solid dysphagia. She denied neck trauma. On physical examination, there was a tough and painful mass and ecchymosis in the thyroid bed. Nasofiberoscopy showed bulging of the left lateral pharyngeal wall leading to right displacement of the endolarynx. Left ventricular fold and ventricle exhibited a violaceous coloration. Computed tomography revealed a nonenhancing collection in the left parapharyngeal space (Figure 1). Magnetic resonance imaging confirmed the presence of a parapharyngeal haematoma with probable origin in a parathyroid adenoma (Figure 2). Analytically, there was parathyroid hormone elevation (242.9 pg/mL, with normal values ranging from 10 to 60 pg/mL). The patient began intravenous methylprednisolone (1 mg/kg/day). After one week, there was complete symptom resolution and fiberoscopy showed neither pharyngeal bulging nor endolaryngeal displacement (Figure 3).

## 3. Discussion

This is a very rare clinical situation: until 2009, only 28 cases of spontaneous cervical haematoma caused by extracapsular rupture of the parathyroid gland were reported [4]. Expansion and bleeding outside the capsule may be due to the fact that parathyroid glands containing tumors have relatively thin and weak capsules [5]. The thyroid and the parathryoid glands are wrapped in a part of the pretracheal fascia, which is deficient on the posterior aspect of the isthmus and most of the posteromedial surface of the lobe and so extends laterally to blend with the carotid sheath and the prevertebral fascia. Consequently, bleeding from those glands can spread to the retropharyngeal and parapharyngeal spaces [3]. Mismatch between growing of the gland and vascular supply may lead to infarction and thus necrosis and bleeding [6]. Acute neck swelling, ecchymosis of neck and chest, and parathyroid hormone elevation are three characteristic criteria for hemorrhaging parathyroid tumors [5]. The majority of patients remain stable and may be treated conservatively or with planned surgical interventions [2, 5, 6]. In these cases, some authors state that surgery should be performed more than 3 months after the occurrence of hemorrhage because the dissection then becomes as simple as for any other form of planned surgery [5, 7]. However, when there is airway compromise or active bleeding, emergent surgical treatment must be ensued [3, 5, 6].

## 4. Conclusion

Many conditions can be responsible for sudden neck swelling. After excluding trauma, blood test results and imaging are crucial for an accurate etiological diagnosis. Close monitoring of the airway is essential, in order to do emergent treatment in life-threatening cases.
